# Assessment of Lower-limb Vascular Endothelial Function Based on Enclosed Zone Flow-mediated Dilation

**DOI:** 10.1038/s41598-018-27392-3

**Published:** 2018-06-18

**Authors:** Harutoyo Hirano, Renjo Takama, Ryo Matsumoto, Hiroshi Tanaka, Hiroki Hirano, Zu Soh, Teiji Ukawa, Tsuneo Takayanagi, Haruka Morimoto, Ryuji Nakamura, Noboru Saeki, Haruki Hashimoto, Shogo Matsui, Shinji Kishimoto, Nozomu Oda, Masato Kajikawa, Tatsuya Maruhashi, Masashi Kawamoto, Masao Yoshizumi, Yukihito Higashi, Toshio Tsuji

**Affiliations:** 1 Academic Institute, College of Engineering, Shizuoka University, Hamamatsu, 739-8527 Japan; 2 Graduate School of Engineering, Hiroshima University, Higashi-Hiroshima, 739-8527 Japan; 3 Department of System Cybernetics, Faculty of Engineering, Hiroshima University, Higashi-Hiroshima, 739-8527 Japan; 4 Nihon Kohden corporation, Tokorozawa, 359-8580 Japan; 5 Department of Anesthesiology and Critical Care, Graduate School of Biomedical and Health Sciences, Hiroshima University, Hiroshima, 734-8553 Japan; 6 Department of Cardiovascular Medicine, Graduate School of Biomedical and Health Sciences, Hiroshima University, Hiroshima, 734-8553 Japan; 7 Department of Cardiovascular Regeneration and Medicine, Research Institute for Radiation Biology and Medicine, Hiroshima University, Hiroshima, 734-8553 Japan; 8 Department of Cardiovascular Physiology and Medicine, Graduate School of Biomedical and Health Sciences, Hiroshima University, Hiroshima, 734-8553 Japan; 9 Division of Regeneration and Medicine, Medical Center for Translational and Clinical Research, Hiroshima University Hospital, Hiroshima, 734-8551 Japan

## Abstract

This paper proposes a novel non-invasive method for assessing the vascular endothelial function of lower-limb arteries based on the dilation rate of air-cuff plethysmograms measured using the oscillometric approach. The principle of evaluating vascular endothelial function involves flow-mediated dilation. In the study conducted, blood flow in the dorsal pedis artery was first monitored while lower-limb cuff pressure was applied using the proposed system. The results showed blood flow was interrupted when the level of pressure was at least 50 mmHg higher than the subject’s lower-limb systolic arterial pressure and that blood flow velocity increased after cuff release. Next, values of the proposed index, %*ezFMD*_L_, for assessing the vascular endothelial function of lower-limb arteries were determined from 327 adult subjects: 87 healthy subjects, 150 subjects at high risk of arteriosclerosis and 90 patients with cardiovascular disease (CAD). The mean values and standard deviations calculated using %*ezFMD*_L_ were 30.5 ± 12.0% for the healthy subjects, 23.6 ± 12.7% for subjects at high risk of arteriosclerosis and 14.5 ± 15.4% for patients with CAD. The %*ezFMD*_L_ values for the subjects at high risk of arteriosclerosis and the patients with CAD were significantly lower than those for the healthy subjects (*p* < 0.01). The proposed method may have potential for clinical application.

## Introduction

Arteries are composed of three layers: the tunica aventitia, the tunica media and the tunica intima. The vascular endothelium is the innermost layer of the tunica intima, and consists of monostratal vascular endothelial cells that produce various vasodilators such as nitric oxide (NO) to adjust for the coarctation of vascular vessels and maintain flexibility^1^. When vascular endothelial function deteriorates as a result of risk factors such as diabetes and hypertension, the flexibility of vascular vessels becomes impaired and arteriosclerosis develops^2,3^. Arteries in the lower-limbs are sites of predilection for peripheral artery disease (PAD), which is a type of advanced arteriosclerosis^4^ caused by vascular endothelial dysfunction and can result in subcutaneous tissue necrosis if treatment is delayed. Arteriosclerosis of the lower-limb arteries is reportedly related to the progression of systemic cardiovascular conditions such as coronary arterial disease and carotid stenosis^5^. Accordingly, quantitative assessment of vascular endothelial function in these arteries may support the early recognition and treatment of arteriosclerosis.

Numerous methods for assessing vascular endothelial function in lower-limb arteries have been proposed. As an example of an invasive approach, blood flow variations were measured using plethysmography after the patient was given a NO agonist drug or a NO antagonist drug^6,7^. However, invasive methods carry risk, cause patient discomfort and are problematic to apply because a NO agonist drug or a NO antagonist drug must be delivered arterially via a catheter. Meanwhile, flow-mediated dilation (FMD) is often used as a non-invasive method for assessing upper-limb vascular endothelial function^8,9^. FMD allows vascular endothelial function evaluation based on vascular diameter variations measured with an ultrasound device. In FMD testing, shear stress caused by increased blood flow after arterial avascularization and release applies a stimulus to the vascular endothelium, which in turn produces NO. However, the test requires an ultrasonologist with a high level of technical ability for accurate measurement of the patient’s vascular diameter accurately over an extended period, and its results depend on the patient’s blood pressure^10^. In addition, it is difficult to accurately measure the vascular diameter of lower-limb arteries with an ultrasound device because they are located deep under the skin and ultrasonic signals do not propagate well through biological tissues. It is therefore technically challenging to apply FMD testing to lower-limb arteries. With this in mind, the our research group proposed a novel method for determining the vascular endothelial function of upper-limb arteries easily without the use of an ultrasound device^11–13^. This technique, known as the enclosed zone FMD (ezFMD), involves the application of oscillometry (as used with commercial automated sphygmomanometers at patients’ homes and in hospitals) rather than vascular diameter measurement using an ultrasound device. In the ezFMD approach, plethysmograms are extracted using an air cuff attached to an upper limb, and the vascular endothelial function of the upper-limb arteries is assessed by calculating the difference in the maximum amplitudes of the extracted plethysmograms before and after cuff occlusion. The ezFMD is a noninvasive and simple method for assessing endothelial function compared to the FMD. Our previous studies showed that the ability of ezFMD to assess endothelial function at the upper arm was equal to or greater than that of FMD at the forearm and has good potential for screening with minimal technical requirements to assess endothelial function because there was wide variability in both ezFMD and FMD^12,13^. However, the ezFMD method has not been applied to lower-limb arteries, which are sites of predilection for arteriosclerosis.

Therefore, the authors propose a novel noninvasive and easy method for assessing the vascular endothelial function of lower limb arteries based on the ezFMD approach, which does not use an ultrasound device.

## Methods

### Subjects

A total of 327 adults (236 males, 91 females; mean age ± S.D.: 55.9 ± 21.2 yrs) were used for the lower-limb ezFMD measuring experiment. The subject breakdown is as follows: 87 healthy subjects (78 males and 9 females; mean age ± S.D.: 30.8 ± 14.9 yrs), 150 subjects at high risk of arteriosclerosis (90 males and 60 females; mean age ± S.D.: 62.0 ± 16.2 yrs) and 90 patients with cardiovascular disease (CAD) (68 males and 22 females; mean age ± S.D.: 70.2 ± 10.3 yrs). Healthy subjects had no history of cardiovascular diseases, liver diseases, renal diseases, autoimmune diseases, or malignant diseases and had no coronary risk factors, including hypertension, dyslipidemia, diabetes mellitus, and current smoking. The subjects at high risk of arteriosclerosis were defined as people who were affected by one or more of the following: hypertension, diabetes, dyslipidemia, or current cigarette smoking. Hypertension was defined as systolic blood pressure of >140 mmHg or diastolic blood pressure of > 90 mmHg, in a sitting position, on at least three different occasions^14^. Diabetes mellitus was defined according to the American Diabetes Association^15^. Dyslipidemia was defined according to the third report of the National Cholesterol Education Program^16^. Current smokers were defined as smokers who had smoked ≥ 1 pack-year, 1 pack-year being defined as 20 cigarettes per day for 1 year. CAD was defined as coronary artery disease, cerebrovascular disease, and PAD. Coronary artery disease included angina pectoris, myocardial infarction, and unstable angina. Cerebrovascular disease included ischemic stroke, hemorrhagic stroke, and transient ischemic attack. Patients with intermittent claudication or revascularization (surgery or catheter-based interventions), or limb amputation were diagnosed as PAD. Framingham risk score was calculated with points for the following risk factors: age, total cholesterol level, high-density lipoprotein cholesterol level, systolic blood pressure, and smoking status^17^. Estimated glomerular filtration rate was calculated using the following equation: 194 × serum creatinine^−1.0949^ × age^−0.287^ (× 0.739 for women)^18^.

In addition, the 8 males of the above healthy subjects (mean age ± S.D.: 23.1 ± 0.8 yrs) were randomly chosen for measuring the lower-limb blood flow and the other 5 males of the above healthy subjects (mean age ± S.D.: 22.2 ± 0.8 yrs) were randomly selected to measure the lower-limb ezFMD of continuous days. The FMD testing was performed for 89 randomly chosen subjects: 8 healthy subjects (6 males and 2 females; mean age ± S.D.: 38.2 ± 16.1 yrs), 49 subjects at high risk of arteriosclerosis (33 males and 16 females; mean age ± S.D.: 60.2 ± 16.1 yrs), and 32 patients with CAD (29 males and 3 females; mean age ± S.D.: 66.1 ± 11.1 yrs).

Experiments were conducted in accordance with the Declaration of Helsinki. Informed consent was obtained from all study participants before the experiments were performed and the study was approved by the ethics committee of Hiroshima University (https://upload.umin.ac.jp. Unique identifier: UMIN000004902).

### Study Protocol

The proposed system requires fulfillment of the following four criteria for application of ezFMD to lower-limb arteries:

1. The system can be used to perform lower-limb arterial blood flow occlusion and prompt post-occlusion reactive hyperemia.
2. The proposed method’s evaluation index %*ezFMD*_*L*_ is highly reproducible.
3. The %*ezFMD*_*L*_ index shows statistically significant differences between healthy subjects and subjects at high risk of arteriosclerosis and between healthy subjects and patients with cardiovascular disease.
4. The %*ezFMD*_*L*_ index shows a significant correlation to %*ezFMD*_*B*_ (the index for evaluating vascular endothelial function at the brachial artery) for the same healthy subjects because these subjects are considered to have normal vascular endothelial function in all arteries.

#### Lower-limb blood flow measuring experiment

Blood flow was observed during lower-limb ezFMD measurement to confirm the fulfillment of requirement (1). The target arteries for evaluation of vascular endothelial function in terms of lower-limb ezFMD are the anterior tibial artery, the posterior tibial artery and the fibular artery. However, as it is difficult to observe blood flow in these arteries with ultrasound due to their location deep below the skin, flow in the dorsal pedis artery (located in the periphery of the anterior tibial artery) was observed instead. Blood flow volume *F*_*b*_(*t*) is often measured for observation of reactive hyperemia. The equation used here is:

1 $${F}_{b}(t)=\frac{\pi }{4}{V}_{b}(t)\{{D}_{b}(t{)\}}^{2},$$

where *V*_*b*_(*t*) and *D*_*b*_(*t*) are blood flow velocity and vascular diameter, respectively. Vascular diameter needs to be measured to determine blood flow volume *F*_*b*_(*t*) as Equation (1). However, it is technically difficult to track immediate variations in vascular diameter with a Doppler ultrasound blood flowmeter based on the echo tracking method while measuring cuff oscillation and during the period immediately after the release of cuff occlusion. It was therefore verified that the proposed system can be used to perform blood flow occlusion and prompt post-occlusion reactive hyperemia in lower-limb arteries.

The chosen 8 healthy subjects were instructed to abstain from eating for at least eight hours beforehand to eliminate the effects of numerous factors that influence vasodilatation^8^. All subjects were instructed to assume a resting prone position for five minutes before blood flow velocity was measured. The velocity *V*_*b*_(*t*) in the dorsal pedis artery was measured with a Doppler ultrasound blood flowmeter (QFM-21, Hadeco) while lower-limb ezFMD was measured with an ezFMD measuring device (FMD tester, Nihon Kohden). Blood flow velocity *V*_*b*_(*t*), cuff pressure and oscillation were measured at 1,000 Hz using an analog-to-digital converter (CSI-360116, Interface), and the data obtained were saved to a PC. The root mean square (RMS) of blood flow velocity is calculated to evaluate blood flow variations, and RMS values calculated using the following equation from data collected before, during and after cuff occlusion were compared:

2 $${V}_{brms}=\sqrt{\frac{1}{{T}_{2}-{T}_{1}}{\int }_{{T}_{1}}^{{T}_{2}}{\{{V}_{b}(t)\}}^{2}dt},$$

where *T*_1_, and *T*_2_ are the RMS calculation start and end times, respectively. The time ranges for calculation were 30 seconds before occlusion, 3 minutes during occlusion and 30 seconds immediately after occlusion. Bonferroni correction was applied for comparison of the means of all subjects’ RMS values calculated from data collected before, during and after cuff occlusion.

#### Lower-limb ezFMD measuring experiment

Lower-limb ezFMD was measured using the protocol outlined in Methods Chapter to verify requirements (2)–(4). Blood pressure in these arteries was measured with a physiological monitor (OPV1510, Nihon Kohden), and an ezFMD measuring device was used to measure oscillation and apply occlusion. The cuff pressure and oscillation were measured at 1000 Hz using an analog-to-digital converter and saved to a PC.

Paired *t*-testing was first performed to compare the mean of oscillation amplitudes in data collected before and after cuff occlusion. The testing was conducted for each mean value for the healthy subjects and the subjects at high risk of arteriosclerosis and those with CAD. Tukey-Kramer testing was also applied to compare the means calculated from all subjects’ %*ezFMD*_*L*_ values among the healthy subjects and the subjects at high risk of arteriosclerosis and those with CAD to verify requirement (3).

Next, the %*ezFMD*_*L*_s of the selected 5 healthy males were measured on five consecutive days to verify requirement (2). The subjects were instructed to abstain from eating for at least eight hours before each measurement. The coefficient of variation value *CV* was calculated using %*ezFMD*_*L*_ data collected over the five days, and the mean *CV* value for the five subjects was also calculated. *CV* value was determined using the following equation:

3 $$CV=\frac{SD}{\bar{x}},$$

where *SD* and $\bar{x}$ are the standard variation and mean, respectively, of the %*ezFMD*_*L*_ values measured over the five days. To evaluate the proposed system’s capacity for repeatability, the mean *CV* value for the lower-limb ezFMD measured from the five subjects was compared with the corresponding value for the brachial artery as measured from five other healthy males (mean age ± S.D.: 23.2 ± 0.7 yrs) using an ezFMD measuring device designed for this purpose^19^. Welch’s *t*-test was used to compare the mean *CV* value for the lower-limb ezFMD with the brachial values.

There is no index with which %*ezFMD*_*L*_ can be compared to verify the effectiveness of the proposed system because no research group has proposed a non-invasive method for quantitative evaluation of lower-limb vascular endothelial function. However, as healthy subjects are considered to have normal vascular endothelial function in all arteries, a correlation between lower-limb and upper-limb artery function can be assumed (requirement (4)). In this experiment, both the ezFMD of the brachial artery and the lower-limb arterial ezFMD were measured in all subjects, and the measured %*ezFMD*_*L*_ was statistically compared with %*ezFMD*_*B*_^12^ (representing the rate of change in cuff oscillation amplitude in the brachial artery as determined using an ezFMD measuring device designed for this purpose). Here, upper-limb blood pressure was measured with a physiological monitor. Cuff pressure and oscillation were measured at 1,000 Hz using an analog-to-digital converter and saved to a PC. The correlation coefficient and regression lines were calculated from each subject’s %*ezFMD*_*L*_ and %*ezFMD*_*B*_ to verify requirement (4). Testing for no correlation was conducted to evaluate the calculated correlation coefficient. In addition, receiver operating characteristic (ROC) analysis^20^ was performed for the calculated %*ezFMD*_*L*_ and %*ezFMD*_*B*_ values to evaluate lower-limb vascular endothelial function screening capacity. %*FMD*, the de facto standard index for estimating vascular endothelial function, and the ankle brachial pressure index^21^ were also measured with an ultrasound device for FMD testing (UNEXEF-18G, Unex) and a blood pressure pulse wave inspection device (form PWV/ABI BP-203, Omron Colin), respectively.

### Lower-limb vascular endothelial function assessment method

Intravascular pressure and external pressure originating from the cuff of the automated sphygmomanometer are applied to the arterial wall while blood pressure is measured. The maximum variation of the cuff’s plethysmogram signals, which shows the difference between systolic and diastolic pressures, is determined under a condition in which intravascular pressure is approximately equal to the external pressure originating from the cuff because arterial wall compliance is maximized in this state.

Cuff plethysmogram signals are related to vascular vessel volume. Assuming that the temperature in the cuff is constant, the relationship between the internal cuff volume *V* and the internal cuff pressure *P* can be expressed as

4 $$P\times V=const\mathrm{.,}$$

where the vascular volume increases by Δ*V*, the cuff volume decreases by Δ*V* and the cuff pressure increases by Δ*P* in relation to cuff oppression caused by increased cuff volume. The oscillation amplitude Δ*P* can be expressed as

5 $$(P+{\rm{\Delta }}P)\times (V-{\rm{\Delta }}V)=P\times V\mathrm{.}$$

Assuming that Δ*P* × Δ*V* is minute compared with the other terms, Equation (5) can be described as

6 $${\rm{\Delta }}P=\frac{{\rm{\Delta }}V\times P}{V}\mathrm{.}$$

Therefore, if the cuff volume *V* and the pressure in the cuff *P* are constant, the oscillation amplitude Δ*P* is proportional to the vascular volume variation Δ*V*. Here, vasodilation is evaluated from the oscillation amplitude Δ*P*.

Blood flow increases sharply after the vascular vessel is released from cuff occlusion with a level of pressure higher than that of systolic blood pressure for a given length of time. This physiological phenomenon is called reactive hyperemia^22^. Shear stress is generated at the boundary of the vascular endothelium in relation to the increased blood flow in the area of cuff occlusion. Vascular endothelial cells are stimulated due to the release of various vasodilators such as NO, and this NO causes increased compliance of the vascular smooth muscle in the tunica media. The relationship linking vascular volume variation Δ*V*, pulse pressure *PP* and arterial compliance *C* can be expressed as following^23^:

7 $${\rm{\Delta }}V=C\times PP\mathrm{.}$$

Assuming that blood pressure is equal before and after cuff occlusion, the following Equation (8) can be obtained from Equations (6) and (7):

8 $$\frac{{\rm{\Delta }}{P}_{2}}{{\rm{\Delta }}{P}_{1}}=\frac{{\rm{\Delta }}{V}_{2}}{{\rm{\Delta }}{V}_{1}}=\frac{{C}_{2}}{{C}_{1}},$$

where Δ*V*_1_ and Δ*V*_2_ are the vascular volume variation before and after cuff occlusion, *C*_1_ and *C*_2_ represent arterial compliance before and after cuff occlusion, and Δ*P*_1_ and Δ*P*_2_ show the oscillation amplitude before and after cuff occlusion. Vascular compliance variations can thus be measured by comparing the oscillation amplitude before and after cuff occlusion.

**Figure 1:**
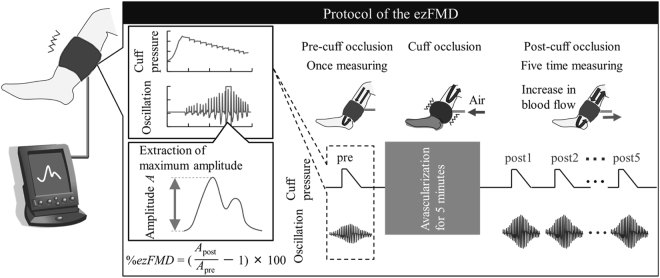
Overview of the proposed lower-limb ezFMD measuring system.

Figure 1 shows the proposed lower-limb ezFMD measurement system, which consists of three parts: (i) a measurement part for determination of cuff oscillation using the oscillometric approach; (ii) an analysis part for determination of vascular feature quantity from the measured oscillation; (iii) an assessment part for evaluation of vascular endothelial function in lower-limb arteries based on the feature quantity determined in (ii).

In the measurement part, the standard cuff used with an oscillometric automatic sphygmomanometer is attached to the ankle of the subject at rest in a prone position. Oscillation measurement and arterial occlusion are performed with the standard cuff and the mean pressure method^24^ result in improved repeatability in the upper limbs during cuff depressurization. Occlusion pressure is adjusted to a level at least 50 mmHg higher than the subject’s systolic arterial pressure so that arteries are completely occluded^8^. Cuff oscillation is first measured once, and occlusion is then applied for five minutes. Cuff oscillation is measured again five times at 30-second intervals after the release of the occlusion.

In the analysis part, the maximum amplitude per beat is determined from each oscillation measurement. With the proposed method, the effects of lower-limb arterial blood pressure are eliminated by applying cuff pressure corresponding to the subject’s blood pressure, and the vascular compliance characteristics of the arterial wall are extracted without the influence of this pressure. The method therefore enables evaluation of the maximum vascular compliance based on calculation to determine beat amplitudes in each oscillation measurement.

In the assessment part, the rate of change in lower-limb arterial amplitude %*ezFMD*_*L*_ is calculated using the maximum amplitudes observed before and after cuff occlusion in the analysis part and the following equation:

9 $$ \% ezFM{D}_{L}=(\frac{{A}_{{\rm{post}}}}{{A}_{{\rm{pre}}}}-1)\times \mathrm{100,}$$

where *A*_pre_ and *A*_post_ are the maximum amplitude before the cuff occlusion and the average of the measured maximum amplitude during the period from the third to the fifth measured oscillations after the cuff occlusion in consideration of vasodilatation response time^25^, respectively. After occlusion, vascular volume variation is considered to increase and oscillation waves are considered to expand because the NO released causes relaxation of the smooth muscle in the tunica media. As a result, if vascular endothelial function is normal, the maximum amplitude will increase after occlusion and the calculated rate of change in amplitude will be positive.

The author thus defined the index %*ezFMD*_*L*_ for the evaluation of vascular dilatation variations before and after occlusion, and the proposed index was used for quantitative evaluation of lower-limb vascular endothelial function.

## Results

### Baseline clinical characteristics

The baseline clinical characteristics of the subjects are summarised in Table 1. The age range was 21–90 years. Of the 327 subjects, 236 (72.2%) were males and 91 (22.3%) were females. 173 (58.1%) had hypertension, 158 (53.0%) had dyslipidemia, 73 (24.5%) had diabetes mellitus and 39 (13.2%) were smokers. The mean value of ezFMD was 23.0 ± 14.5%. No significant difference was found between males and females (23.1 ± 14.9 v.s. 22.9 ± 13.0, *p* = 0.90).

**Table 1:** Clinical characteristics of the Subjects.

Variables	Total (n = 327)
Age, y	55.9 ± 21.2
Body mass index, kg/m^2^	24.0 ± 11.5
Systolic blood pressure, mmHg	133.3 ± 23.2
Diastolic blood pressure, mmHg	62.0 ± 11.0
Heart rate, bpm	66.9 ± 11.9
Ankle brachial pressure index	1.10 ± 0.14
Total cholesterol, mmol/L	182.3 ± 37.8
Triglycerides, mmol/L	121.2 ± 72.8
HDL-C, mmol/L	57.4 ± 16.1
LDL-C, mmol/L	106.6 ± 32.9
Glucose. mmol/L	110.6 ± 43.4
Hypertension, n, %	173 (58.1%)
Dyslipidemia, n, %	158 (53.0%)
Diabetes mellitus, n, %	73 (24.5%)
Smoking, n, %	39 (13.2%)
Coronary heart disease, n, %	63 (21.1%)
Cerebrovascular disease, n, %	26 (8.7%)
Peripheral artery disease, n, %	25 (8.4%)
Framingham risk score, %	15.1 ± 10.8
ezFMD, %	23.0 ± 14.5

All results are presented as mean ± S.D. or number (%).

HDL-C, high-density lipoprotein cholesterol;

LDL-C, low-density lipoprotein cholesterol;

ezFMD, enclosed zone flow-mediated dilation.

### Lower-limb blood flow measuring experiment

**Figure 2:**
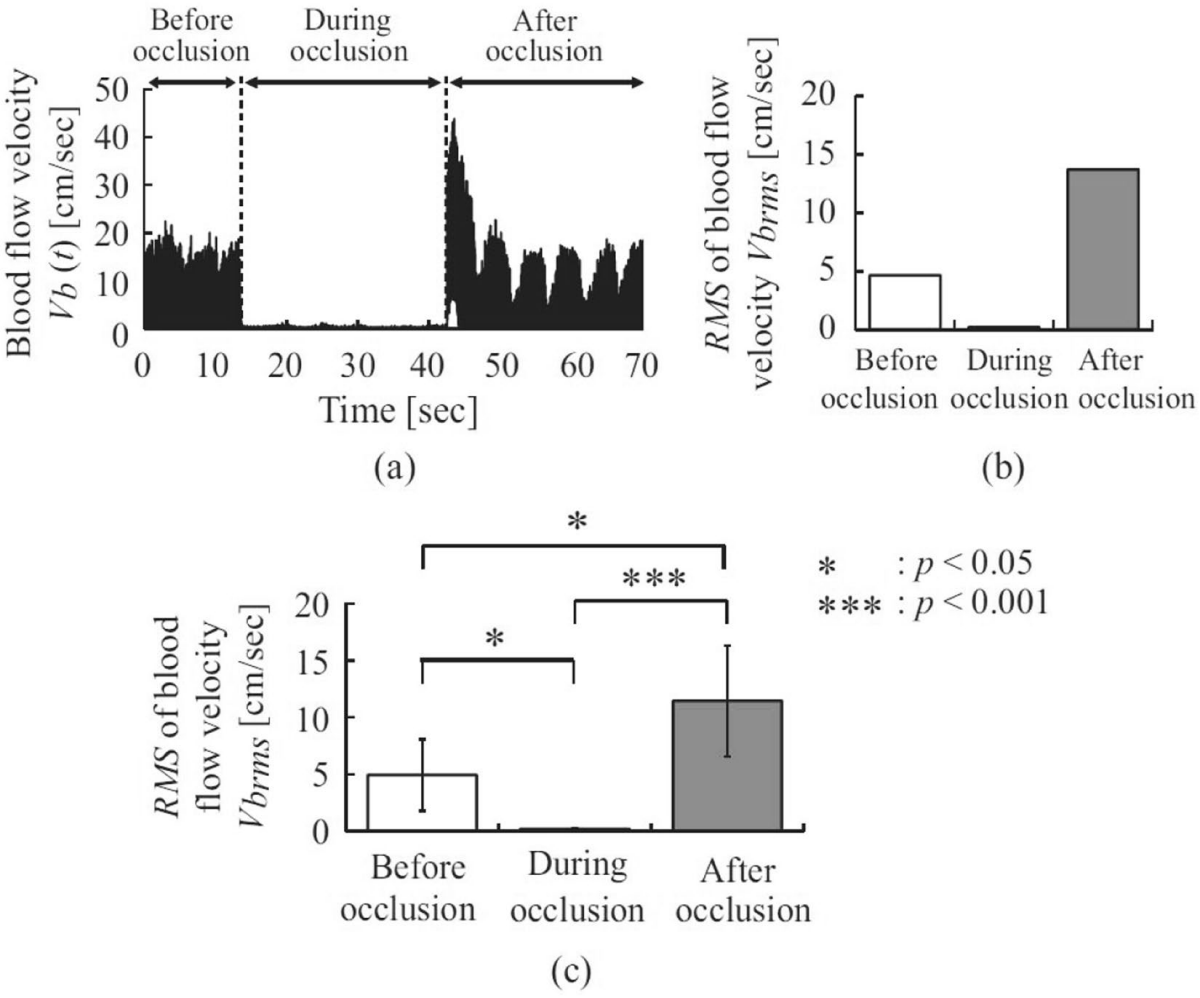
Blood flow velocity during lower-limb ezFMD measurement: (**a**) time variation of blood flow velocity (Sub. A), (**b**) RMS of blood flow velocity (Sub. A), (**c**) RMS of blood flow velocity (all subjects).

Figure 2(a) shows the blood flow velocity *V*_*b*_(*t*) measured while the lower-limb ezFMD of a healthy male subject (Sub. A) was measured. Figure 2(b) shows the calculated RMSs of blood flow velocity ${V}_{{b}_{RMS}}$ before, during and after cuff occlusion as measured from a healthy subject (Sub. A). The measured blood flow velocity *V*_*b*_(*t*) varied during the period before and after cuff occlusion due to the effect of the cuff pressure applied for plethysmogram extraction. Figure 2(a) shows that measured blood pressure *V*_*b*_(*t*) was approximately 0 cm/sec during cuff occlusion and increased sharply immediately afterward. Figure 2(b) shows that the RMS of the measured blood pressure ${V}_{{b}_{RMS}}$ was close to 0 cm/sec during occlusion and increased afterward. Figure 2(c) shows the means and standard deviations of the RMS of measured blood pressure ${V}_{{b}_{RMS}}$ for all subjects. It can be seen that latter value was close to 0 cm/sec during cuff occlusion and significantly increased afterward compared with the corresponding value before occlusion for all subjects (*p* < 0.01).

### Lower-limb ezFMD measuring experiment

**Figure 3:**
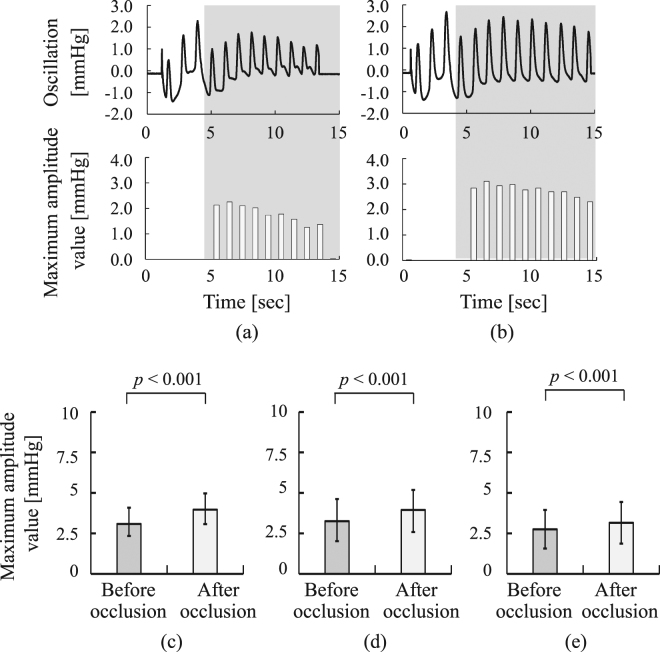
Measured results of cuff oscillation: (**a**) air-cuff oscillation and maximum amplitude measured from a healthy subject (Sub. A) before cuff occlusion, (**b**) those after cuff occlusion, (**c**) comparison of maximum amplitude between before and after cuff occlusion in healthy subjects, (**d**) that in subjects at high risk of arteriosclerosis, (**e**) that in patients with CAD.

Figure 3(a,b) show the measured cuff oscillation waveform and calculated amplitude value from beat to beat before and after cuff occlusion for a healthy male subject (Sub. A), where the amplitude was calculated from the beats within the shaded area. It can be seen that the amplitude was higher after occlusion than before. Figure 3(c–e) show the mean values and standard deviations calculated from the oscillation amplitude before and after cuff occlusion based on measurements from the healthy subjects, subjects at high risk of arteriosclerosis and patients with CAD, respectively. Figure 3(c–e) indicated that the amplitudes after the cuff occlusion were significantly higher than those before the cuff occlusion among the healthy subjects, the subjects at high risk of arteriosclerosis and the patients with CAD (healthy subjects: *p* = 1.2 × 10^−39^, subjects at high risk of arteriosclerosis: *p* = 2.5 × 10^−46^, patients with CAD: *p* = 5.4 × 10^−13^).

**Figure 4:**
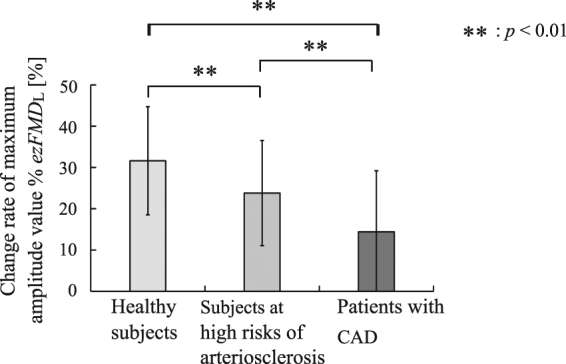
Results of comparison for calculated %*ezFMD*_*L*_ among healthy subjects, subjects at high risk of arteriosclerosis and patients with CAD.

Figure 4 shows that the mean values and standard deviations calculated from %*ezFMD*_*L*_ for the healthy subjects, the subjects at high risk of arteriosclerosis and those with CAD were 30.5 ± 12.0%, 23.6 ± 12.7% and 14.5 ± 15.4%, respectively. Significant differences were confirmed between the %*ezFMD*_*L*_ of the healthy subjects and that of the subjects at high risk of arteriosclerosis, between that of the healthy subjects and that of the patients with CAD and between that of the subjects at high risk of arteriosclerosis and that of the patients with CAD (healthy subjects v.s. subjects at high risk of arteriosclerosis: *p* = 4.1 × 10^−5^, healthy subjects v.s. patients with CAD: *p* = 1.0 × 10^−12^, subjects at high risk of arteriosclerosis v.s. patients with CAD: *p* = 5.7 × 10^−6^). The mean values and standard deviations of measured %*FMD* for the healthy subjects, subjects at high risk of arteriosclerosis, and those with CAD were 7.83 ± 4.42%, 4.81 ± 4.03% and 3.43 ± 2.27%, respectively. A significant difference was confirmed between the healthy subjects and the patients with CAD (*p* = 2.6 × 10^−2^), whereas no significant differences were confirmed between the healthy subjects and those at a high risk of arteriosclerosis or between those at a high risk of arteriosclerosis and those with CAD (healthy subjects vs. those at high risk of arteriosclerosis: *p* = 1.0 × 10^−1^, subjects at high risk of arteriosclerosis vs. patients with CAD: *p* = 5.4 × 10^−2^). In contrast, mean values of %*ezFMD*_*L*_ for the same subjects who underwent FMD testing were 35.8 ± 10.6%, 24.1 ± 12.7%, and 17.6 ± 15.4%, respectively. Significant differences were confirmed among all subject groups (healthy subjects vs. subjects at high risk of arteriosclerosis: *p* = 1.8 × 10^−2^; healthy subjects vs. patients with CAD: *p* = 1.2 × 10^−3^; and subjects at high risk of arteriosclerosis vs. patients with CAD: *p* = 4.9 × 10^−2^).

**Figure 5:**
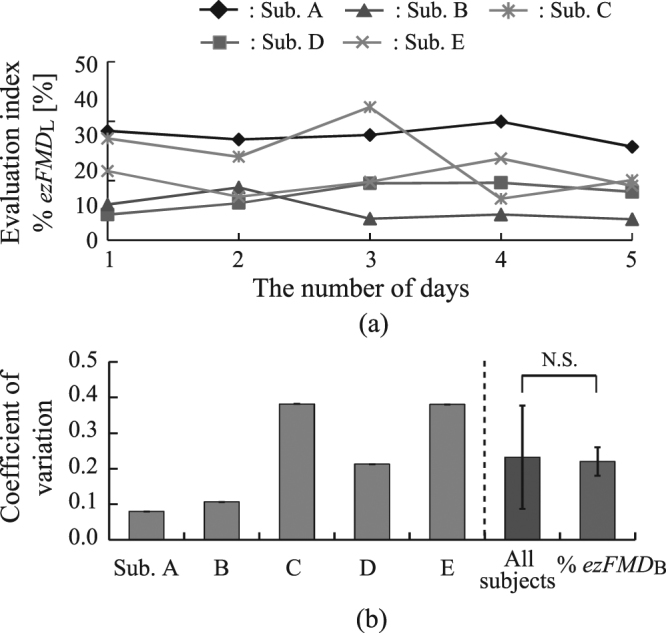
Five-day variation of measured %*ezFMD*_*L*_ in each healthy subject: (**a**) time variations in %*ezFMD*_*L*_, (**b**) comparison of calculated coefficients of variation.

Figure 5(a) shows variations in %*ezFMD*_*L*_ over five consecutive days for all subjects. Figure 5(b) shows that the mean value and standard deviations of CV for the healthy subjects calculated from %*ezFMD*_*L*_ and those of CV measured with the ezFMD measuring device for the brachial artery were 0.23 ± 0.15 and 0.22 ± 0.04, respectively. The CV calculated from %*ezFMD*_*L*_ was thus similar to that from the brachial artery. No statistically significant difference was seen between the CV from %*ezFMD*_*L*_ and that from the brachial artery.

**Figure 6:**
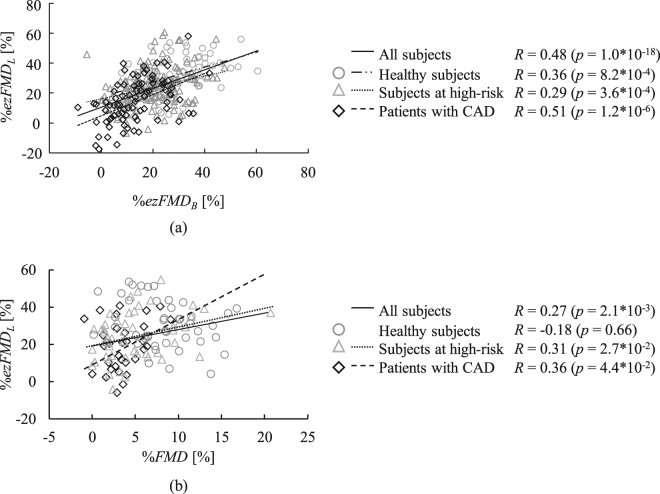
Statistical comparison of indices: (**a**) comparison between the ezFMD of the upper and lower limbs and (**b**) comparison between FMD and ezFMD testing of the lower limbs.

Figure 6(a,b) show the correlation between %*ezFMD*_*B*_ (representing the vascular endothelial function of the brachial artery) and %*ezFMD*_*L*_ and between %*FMD* and %*ezFMD*_*L*_, respectively. The ratio of %*ezFMD*_*L*_ to %*ezFMD*_*B*_ for the healthy subjects, that for the subject at high risk of arteriosclerosis and that for the patient with CAD were 0.48, 0.31, and 0.46, respectively (*p* < 0.001).

**Figure 7:**
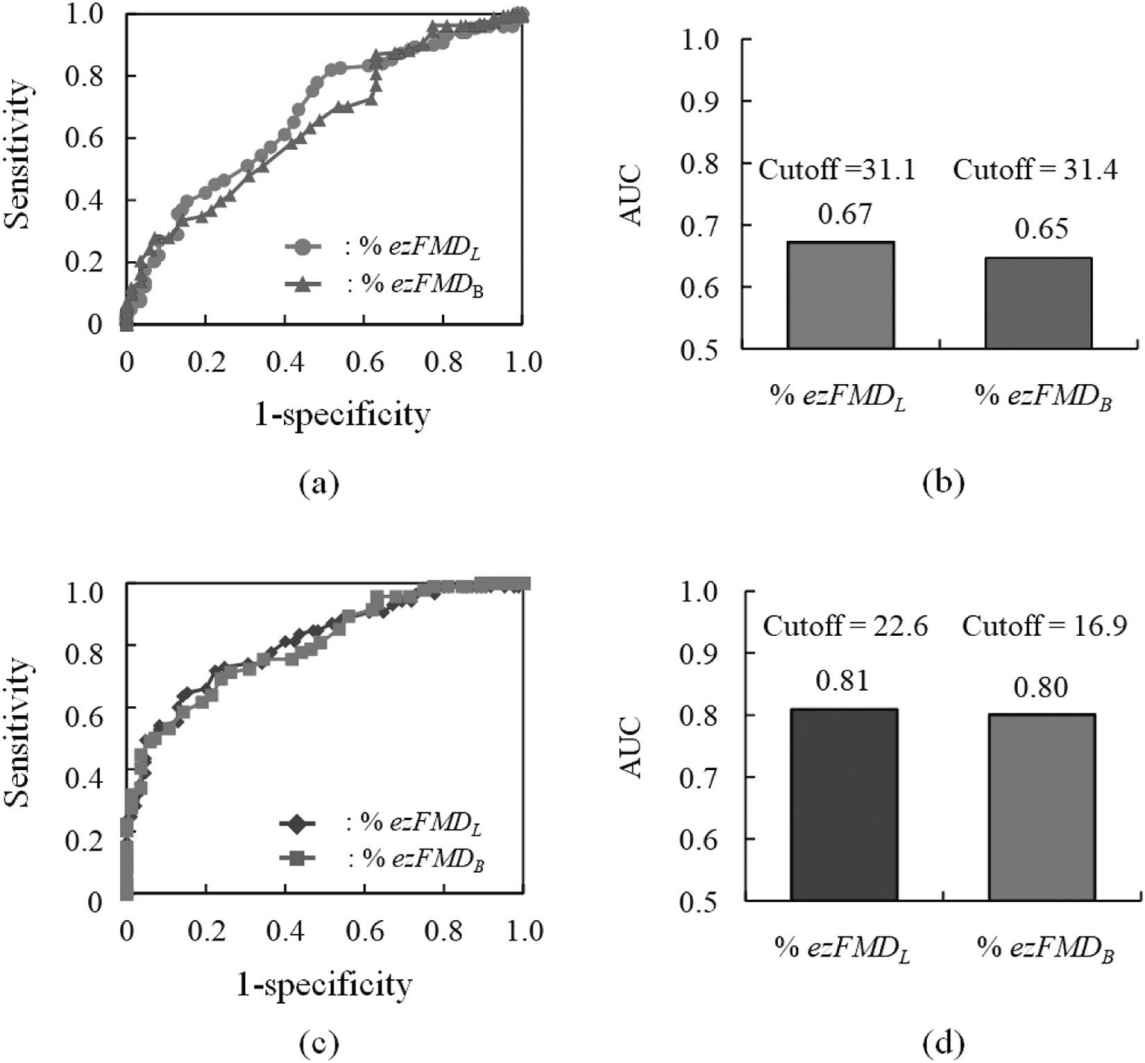
Results of ROC analysis: (**a**) ROC curves for healthy subjects and subjects at high risk of arteriosclerosis, (**b**) calculated AUC of %*ezFMD*_*L*_ and %*ezFMD*_*B*_ for healthy subjects and subjects at high risk of arteriosclerosis, (**c**) ROC curves for healthy subjects and patients with CAD, (**d**) calculated AUC of %*ezFMD*_*L*_ and %*ezFMD*_*B*_ for healthy subjects and patients with CAD.

Figure 7(a,b) show the results of the %*ezFMD*_*L*_ and %*ezFMD*_*B*_ that ROC curve analysis performed to discriminate the subjects at high risk of arteriosclerosis from the healthy subjects and AUCs calculated from the full area under these ROC curves. Figure 7(b) indicates that the AUCs of %*ezFMD*_*L*_ and %*ezFMD*_*B*_ were 0.66 and 0.65, respectively. Figure 7(c,d) show the results of the %*ezFMD*_*L*_ and %*ezFMD*_*B*_ that ROC curve analysis performed to discriminate the patients with CAD from the healthy subjects and AUCs calculated from the full area under these ROC curves. Figure 7(d) indicates that the AUCs of %*ezFMD*_*L*_ and %*ezFMD*_*B*_ were 0.78 and 0.80, respectively.

## Discussion

The blood flow was interrupted by the applied cuff pressure at least 50 mmHg higher than the subject’s lower-limb systolic arterial pressure as shown the measured blood flow velocity *V*_*b*_(*t*) of a healthy male subject (Sub. A) in Fig. 2(a). In addition, shear stress caused by the sharp increase in blood flow velocity *V*_*b*_(*t*) immediately after occlusion release may have caused vascular endothelial cell stimulation. The proposed system is capable of interrupting lower-limb arterial blood flow, as the mean RMS of measured blood pressure ${V}_{{b}_{RMS}}$ during cuff occlusion for all healthy subjects was sufficiently close to 0 cm/sec as shown the mean values and standard deviations of the RMS of measured blood pressure ${V}_{{b}_{RMS}}$ for all healthy subjects in Fig. 2(c). Reactive hyperemia in the lower-limb vascular endothelium is also shown due to the significant increase observed in measured blood flow *V*_*b*_(*t*) after cuff occlusion using the proposed system. These outcomes show that lower-limb blood flow is interrupted and the lower-limb vascular endothelium is stimulated using the proposed system.

The lower-limb arterial dilation after occlusion for a healthy male subject as shown in Fig. 3(a,b). The amplitude values measured from the healthy subjects, the subjects at high risk of arteriosclerosis and the patients with CAD significantly increased just after cuff occlusion release as shown in Fig. 3(c–e). This is because the lower-limb vascular compliance of the healthy subjects increased well after cuff occlusion release in association with sufficient release of NO, and additionally because that of the subjects at high risk of arteriosclerosis and the patients with CAD also increased in association with NO release even though their lower-limb vascular endothelial function may have been weaker than that of the healthy subjects.

The %*ezFMD*_*L*_ of the patients with CAD was significantly lower than that of the healthy subjects and that of the subjects at high risk of arteriosclerosis was significantly lower than that of the healthy subjects as shown in Fig. 4, which satisfies requirement (3) listed in Study Protocol section. This result indicates that the amount of NO produced after cuff occlusion was reduced because of lower-limb vascular endothelial dysfunction.

The CV of %*ezFMD*_*L*_ over a period of five consecutive days exhibited large variations among the healthy subjects as shown in Fig. 5. However, it can be seen that the repeatability of the proposed system is almost equal to that of the previous index *ezFMD*_*B*_. The CV of the proposed system was also better than that of %*FMD*, which is the current de facto standard for non-invasive evaluation of vascular endothelial function. The proposed system therefore satisfied requirement (2) listed in Study Protocol section and may be considered useful in the assessment of lower-limb vascular endothelial function.

The index %*ezFMD*_*L*_ exhibited a moderate correlation with %*ezFMD*_*B*_ in the healthy subjects and the patients with CAD as shown in Fig. 6. These outcomes indicate that %*ezFMD*_*L*_ satisfied requirement (4) listed in Study Protocol section and that ezFMD can be used for quantitative assessment of lower-limb vascular endothelial function.

The effectiveness of using %*ezFMD*_*L*_ to discriminate patients with CAD from healthy subjects was equal to that of %*ezFMD*_*B*_ as shown in Fig. 7. The discrimination method between patients with CAD and healthy subjects using %*ezFMD*_*L*_ for the diagnosis is to use the cutoff value calculated from the ROC analysis because it is an indicator to distinguish between positive and negative test outcomes. A %*ezFMD*_*L*_ value of 22.6% was optimal for distinguishing between patients with CAD and healthy individuals in this study. Peripheral artery disease is known as an occlusive arterial disease that most commonly affects the legs. It is thus important to measure the vascular endothelial function at the lower-limb arteries. the proposed system is highly promising for assessment to discriminate lower-limb vascular endothelial dysfunction from normal vascular endothelial function in lower-limb arteries.

In conclusion, this paper proposes a novel system for evaluating vascular endothelial function in lower-limb arteries based on ezFMD. The results reported that the system is capable of prompting reactive hyperemia and stimulating the vascular endothelium in lower-limb arteries. A lower-limb ezFMD measuring experiment was conducted on healthy subjects whose vascular endothelial function was normal. The results showed that the measured amplitude of cuff oscillation was significantly higher after cuff occlusion than before for all healthy subjects, and that the repeatability of the proposed index for assessing vascular endothelial function was equal to or better than that of the previous index. The proposed index showed a significant correlation with that of the brachial artery. As the system outlined here has high discrimination ability for lower-limb vascular endothelial dysfunction and lower-limb arteriosclerosis, it was considered effective.

## Study Limitations

The discrimination accuracy between the healthy group and the risk factor group using %*ezFMD*_*L*_ requires further investigation for use as a diagnostic marker. Nitric oxide bioavailability testing was not performed in this study. The assessment of NOx, a marker of nitric oxide bioavailability^26^, would provide more information about ezFMD functionality. The currently proposed system for measuring lower-limb ezFMD has high variability. An improved system for measuring the vascular endothelial function of the lower-limb artery with high accuracy is required.

## References

[1] Vanhoutte PM (1989). Endothelium and control of vascular function. Hypertension.

[2] Ross R (1979). The pathogenesis of atherosclerosis. Mechanisms of Ageing and Development.

[3] Ross R (1999). Atherosclerosis - inflammatory of disease. New England Journal of Medicine.

[4] Society for Vascular Surgery Lower Extremity Guidelines Writing Group *et al*. Society for vascular surgery practice guidelines for atherosclerotic occlusive disease of the lower extremities: Management of asymptomatic disease and claudication. *Journal of Vascular Surgery* **61**, 2S–41S. 10.1016/j.jvs.2014.12.009 25638515

[5] Kawarada O (2003). Carotid stenosis and peripheral artery disease in japanese patients with coronary artery disease undergoing coronary artery bypass grafting. Circulation Journal.

[6] Sanada H (2005). Vascular function in patients with lower extremity peripheral arterial disease: a comparison of functions in upper and lower extremities. Atherosclerosis.

[7] Panza JA, Quyyumi AA, J. E. B, Epstein SE (1990). Abnormal endothelium-dependent vascular relaxation in patients with essential hypertension. New England Journal of Medicine.

[8] Corretti MC (2002). Guidelines for the ultrasound assessment of endothelial-dependent flow-mediated vasodilation of the brachial artery. Journal of the American College of Cardiology.

[9] Widlansky ME, Gokce N, J. F. K, Vita JA (2003). The clinical implications of endthelial dysfunction. Journal of the American College of Cardiology.

[10] Maruhashi T (2013). Relationship between flow-mediated vasodilation and cardiovascular risk factors in a large community-based study. Heart.

[11] Ukawa T (2012). Novel non-invasive method of measurement of endothelial function: enclosedzone flow-mediated dilatation (ezfmd). Medical and Biological Engineering and Computing.

[12] Idei N (2013). A novel noninvasive and simple method for assessment of endothelial function: Enclosed zone flow-mediated vasodilation (ezfmd) using an oscillation amplitude measurement. Atherosclerosis.

[13] Morimoto, H. *et al*. Endothelial function assessed by automatic measurement of enclosed zone flow-mediated vasodilation using an oscillometric method is an independent predictor of cardiovascular events. *Journal of the American Heart Association* **5** (2016). 10.1161/JAHA.116.004385 PMC5210444 28003249

[14] The Japanese Society of Hypertension. The japanese society of hypertension committee for guidelines for the management of hypertension: Guidelines for the prevention of arteriosclerotic disease 2014 (2014).

[15] American Diabetes Association (2014). Diagnosis and classification of diabetes mellitus. Diabetes Care.

[16] Expert Panel on Detection, Evaluation, and Treatment of High Blood Cholesterol in Adults. (2001). Executive summary of the third report of the national cholesterol education program (ncep) expert panel on detection, evaluation, and treatment of high blood cholesterol in adults (adult treatment panel iii). Journal of the American Medical Association.

[17] Wilson PWF, Castelli WP, Kannel WB (1987). Coronary risk prediction in adults (the framingham heart study). American Journal of Cardiology.

[18] Matsuo S (2009). Revised equations for estimated gfr from serum creatinine in japan. American Journal of Kidney Diseases.

[19] Tsuji T (2012). Noninvasive evaluation of endothelial function based on dilation rate of integrated air-cuff plethysmogram. Japanese journal of medical instrumentation.

[20] Lasko TA, Bhagwat JG, Zou KH, Ohno-Machado L (2005). The use of receiver operating characteristics curves in biomedical informatics. Journal of Biomedical Informatics.

[21] Winsor T (1950). Influence of arterial disease on the systolic blood pressure gradients of the extremity. American Journal of the Medical Sciences.

[22] Leng GC, Fowkes FG, Donnan PT, Housley E (1993). Reactive hyperemia test in a random sample of the general population. Journal of Vascular Surgery.

[23] Ukawa, T. *et al*. Improvement of novel noninvasive measurement of endothelial function: ezfmd. In *2011 IEEE/SICE International Symposium on System Integration*, 446–451 (2011).

[24] Ukawa T (2012). Repeatability of novel non-invasive measurement of endothelial function: ezfmd. Therapeutic. Research.

[25] Kihara D (2013). Estimation of arterial viscoelastic properties during the flow-mediated dilation test. Transactions of the Society of Instrument and Control Engineers.

[26] Node K (1995). Plasma nitric oxide end products are increased in the ischemic canine heart. Biochemical and Biophysical Research Communications.

